# Diameter of Carbon Nanotube-Directed Self-Assembly of Amphiphilic Block Copolymers

**DOI:** 10.3390/ma12101606

**Published:** 2019-05-16

**Authors:** Zihao Wang, Susu Tao, Yanyan Chu, Xiaoyan Xu, Qinggang Tan

**Affiliations:** School of Materials Science and Engineering, Key Laboratory for Advanced Civil Engineering Materials (Ministry of Education), Tongji University, Caoan Road 4800, Shanghai 201804, China; henry_zihao@foxmail.com (Z.W.); picassu@163.com (S.T.); chuyy1919@163.com (Y.C.); kadxxy@tongji.edu.cn (X.X.)

**Keywords:** cooperative self-assembly, amphiphilic block copolymers, carbon nanotubes, nanocomposites

## Abstract

The cooperative self-assembly of nanoparticles and amphiphilic block copolymers has attracted increasing interests as it offers effective routes to achieve nanocomposite supramolecular structures with desired structure and properties. The incorporation of nanoparticles usually tunes the self-assembly structure of block copolymers, as the copolymer–nanoparticle interactions may change the relative volume ratio of hydrophobic block/hydrophilic block copolymers. It should be noted that the micro-size length and the strong nonpolar feature of carbon nanotubes (CNTs) may cause the block copolymer–CNT interactions to differ from the the block copolymer–nanoparticle interactions. Herein, we show that the diameter of CNTs and the copolymer/CNT ratio have a profound effect on the self-assembly behavior of amphiphilic block copolymers. Upon the addition of carboxylated multi-walled carbon nanotubes (c-MWCNTs, diameter <8 nm,) to the methoxy polyethylene glycol-poly (D,L-lactic acid) (MPEG-PDLLA) solution, it is difficult to observe the c-MWCNTs directly in TEM images. However, it has been found that they form supramolecular nanocomposite structures with MPEG-PDLLA. Moreover, these supramolecular structures transform from core–shell spherical micelles into rod-like micelles and then into large composite aggregates with the increase of the c-MWCNT addition. However, in the case of the addition of c-MWCNTs with a diameter of 30–50 nm, the dispersed c-MWCNTs and spherical core–shell micelles could be observed simultaneously in the TEM images at a low c-MWCNT addition, and then the micelle structure disappeared and only well-dispersed c-MWNTs were observed in TEM images at a high c-MWCNT addition. A possible model was proposed to explain the rule of CNTs participating in the formation of copolymer/CNT nanocomposite structures. It was also shown that as-prepared copolymer/CNT supramolecular nanocomposites could be used as drug carriers, enabling the adjustment of the drug loading and release time.

## 1. Introduction

The self-assembly of amphiphilic block copolymers offers a powerful route to enable the precise fabrication of multifunctional nanostructures [1,2,3,4,5,6]. These self-assembled nanostructures have been extensively studied and used for a range of biomedical applications including bioimaging and controlled drug delivery [7,8,9]. Key considerations of these nanostructures for biomedical applications include their size, morphology and functionalization. One simple and effective method to derive such nanostructures is to incorporate functionalized nanoparticles in the self-assembled nanostructures, forming nanocomposite supramolecular structures to achieve desired structure and properties [10,11,12,13,14,15,16,17]. These nanocomposites can combine the advantages of both components (nanoparticles and amphiphilic block copolymers) and show some new and interesting properties that can be used as nanocarriers for biomedical applications.

Generally, the incorporation of nanoparticles in self-assembled amphiphilic block copolymers can produce an interaction between nanoparticles and block copolymers that may have a significant impact on the hydrophobic interaction of hydrophobic blocks and the relative ratios of hydrophobic blocks/hydrophilic blocks. The change in these parameters caused by the interaction between nanoparticles and block copolymers can affect the self-assembly behaviors and be used to adjust the structures and properties of nanocomposite supramolecular structures [15].

Among the functional nanomaterials, carbon nanotubes (CNTs) have been shown to be potentially useful as medical imaging contrast agents and drug carriers due to their excellent intrinsic physical and chemical properties [18,19,20,21,22]. However, CNTs have a strong nonpolar feature that makes them poorly dispersive in physiological media and limits their use in biological applications [23,24,25]. Generally, a suitable modification for CNTs needs to be designed to resolve their poor dispersion for biological and biomedical applications [26,27,28,29]. Block copolymers have been successfully used to disperse CNTs in various solutions [30,31,32,33,34,35]. According to the strength of interaction between CNTs and polymers, two modes of interaction have been proposed [36]—one is wrapping mode (strong CNT–polymer interaction) [37,38,39], and the other is non-wrapping mode (weak CNT–polymer interaction) [40,41]. In the non-wrapping mode, when the amphiphilic block copolymers and CNTs mixed in an organic solvent are dialyzed against deionized water, a usually accepted dispersion mechanism in which the hydrophobic blocks of copolymers physically adsorb onto the surface of CNTs and screen the hydrophobic interactions among the CNTs, while the hydrophilic blocks dangle out of the CNTs, providing steric repulsion that causes the good dispersion of CNTs in an aqueous solution. It should be noted that CNTs have two dimensions—the width is nanoscopic and the length is mesoscopic. This size feature of CNTs allows two scenarios in terms of the interactions between CNTs and the hydrophobic blocks of copolymers. One is that hydrophobic block chain of the block copolymers is attached to the surface of CNTs along the diameter (end attachment). The other is that the hydrophobic block chain is adsorbed and arranged on the surface of CNTs along the length.

In the case of surface tether by the ends of the hydrophobic block chain of amphiphilic block copolymers, the amphiphilic block copolymers may form an adsorbed layer on the surface of CNTs known as a polymer brush. However, it should be noted that if the diameter of the CNTs is much smaller than the hydrophobic segment length of the amphiphilic copolymers, most of the hydrophobic chain may dangle outside the CNTs along with the hydrophilic chain. In this case, the amphiphilic block copolymer end-tethered CNTs will constitute a new “temporary shape amphiphile”. This new building block may cooperatively assemble with amphiphilic block copolymers into energetically stable nanocomposite supramolecular structures in an aqueous solution. The formation of “temporary shape amphiphiles” is determined by the size of the diameter of CNTs and the length of the hydrophobic blocks of amphiphilic copolymers. In addition, the copolymer/CNT ratio can also adjust the amphiphilicity of these “temporary shape amphiphiles” that would regulate cooperative self-assembly processes overall. Thus, it is of great interest to explore the effect of the diameter of CNTs and the copolymer/CNT ratio on the formation of supramolecular nanocomposite structures toward the controllable fabrication of functional nanostructures for biomedical applications.

In this paper, a block copolymer, methoxy polyethylene glycol-poly (D,L-lactic acid), and carboxylated multi-walled carbon nanotubes (c-MWCNTs) with varying diameters were used to explore the effects of the diameter of CNTs and the copolymer/CNT ratio on the formation of supramolecular nanocomposites structures. The change in morphology and size of the supramolecular nanocomposite structures were studied in detail by TEM and dynamic light scattering (DLS). Based on the experimental results obtained, a possible explanation for the rule of CNTs participating in the formation of copolymer/CNT supramolecular nanocomposites structures was developed. A representative hydrophobic chemotherapeutic drug, doxorubicin (DOX), was also used to investigate the drug loading and in vitro release behavior of as-prepared copolymer/CNT supramolecular nanocomposite structures in phosphate buffer solution (PBS).

## 2. Materials and Methods

### 2.1. Materials

Monomethoxy poly (ethylene glycol)-block-poly (D,L-lactide) (MPEG-PDLLA, 2K-10K) was obtained from Jinan Daigang Biological Technology Company (Jinan, China). Carboxylated multi-walled carbon nanotubes (c-MWCNTs, diameter <8 nm, length 0.5~2 μm; diameter 30~50 nm, length 0.5~2 μm, –COOH content = 1.23 wt.%) were obtained from Nanjing Xianfeng Nano-Material Technology Company (Nanjing, China). Doxorubicin hydrochloride (DOX HCl) was obtained from Beijing Huafeng United Technology Company (Beijing, China). Dimethyl sulfoxide (DMSO) was obtained from Sinophaim Chemical Reagent Company (Shanghai, China). Dialysis tubes (molecular weight cut off = 8000–12,000) and phosphate buffer solution (PBS) were obtained from Shanghai Yuanye Biological Technology Company (Shanghai, China). All solvents and reagents were of analytical grade and used as such without further purification.

### 2.2. Methods

#### 2.2.1. Preparation of MPEG-PDLLA/c-MWCNT Nanocomposites

MPEG-PDLLA/c-MWCNT nanocomposites were prepared by a membrane dialysis method. Briefly, 10.0 mg DOX∙HCl and 0.5 mL triethylamine were co-dissolved in 5 mL of DMSO and then the mixed solution was stirred overnight to remove the hydrochloride of DOX∙HCl. Subsequently, 40.0 mg MPEG-PDLLA and different amounts of c-MWCNTs were added into the solution (the mass ratio of c-MWCNTs to MPEG-PDLLA was changed from 0:1 to 1:0 gradually). Next, the mixtures were stirred at room temperature for 2 h to form a uniform dispersion. Then, the mixed solutions were transferred to a dialysis tube (molecular weight cut off = 8000–12,000) and dialyzed against deionized water for 48 h to remove the organic solvent. Finally, the expected MPEG-PDLLA/c-MWCNT nanocomposites were obtained. Different MPEG-PDLLA/c-MWCNT nanocomposites were prepared by varying the ratio of copolymer/CNTs and the tube diameter of CNTs.

#### 2.2.2. Characterization of MPEG-PDLLA/c-MWCNT Nanocomposites

A JEOL JEM-2010 microscope was used to obtain transmission electron microscopy (TEM) images of MPEG-PDLLA/c-MWCNT nanocomposites. The TEM was operated at an accelerating voltage of 100 KV. One drop of nanocomposite solution was placed onto a 200 mesh carbon-coated copper grid to make TEM samples. Then the samples were air-dried overnight at room temperature before observation. The average particle size of nanocomposite was determined by averaging the size of more than 100 nanoparticles using image software on TEM images. High-resolution transmission electron microscopy (HRTEM) was carried out using a JEM-2100F high-resolution transmission electron microscope 350 operated at an accelerating voltage of 200 KV.

The particle size distribution of MPEG-PDLLA/c-MWCNT nanocomposites was also determined at 25 °C by a Zetasizer Nano ZS90 instrument (Malvern Instruments) equipped with a multipurpose autotitrator (MPT-2) at a fixed scattering angle of 90° and the results were evaluated using the intensity distribution.

Thermal gravimetric analyses (TGA) of MPEG-PDLLA/c-MWCNT nanocomposites were conducted using a NETZSCH STA 449C instrument at a heating rate of 10 °C/min from 30 °C to 800 °C in a nitrogen atmosphere (flow rate of 60 cm^3^/min).

The dispersion stability of MPEG-PDLLA/c-MWCNT nanocomposites in the PBS (pH 7.4) was also determined using a digital camera at 37 °C.

For drug entrapment measurement, a known weight of DOX-loaded MPEG-PDLLA/c-MWCNT nanocomposites was dissolved in 1 mL of DMSO. The DOX loading content of nanocomposites was measured at an absorbance wavelength of 480 nm on an ultraviolet-visible (UV-vis) spectrophotometer (UV-VIS SP-752 PC spectrophotometer). The drug entrapment (DE%) was defined as follows:

DE% = (weight of DOX loaded in nanocomposites)/(total weight of nanocomposites) × 100%.(1)

#### 2.2.3. In Vitro Drug Release

A 2.0 mL aqueous dispersion of DOX-loaded MPEG-PDLLA/c-MWCNT nanocomposites (2.0 g/L) was introduced into a dialysis tube (molecular weight cut off = 8000–12,000), and then dialyzed against 5 mL of PBS buffer solution (pH 7.4, 10 mM) using a shaker at a speed of 150 rpm for 30 days. An in vitro release study was performed in the dark and at 37 °C simulated human temperature. Subsequently, 5 mL of liquid outside the dialysis bag (PBS buffer solution) was withdrawn at specific time intervals and an equal volume of fresh release medium was added again. The amounts of released DOX from the nanocomposites were determined using UV-vis spectrophotometry at 480 nm.

## 3. Results and Discussion

### 3.1. Characterization of MPEG-PDLLA/c-MWCNT Nanocomposites

**Figure 1:**
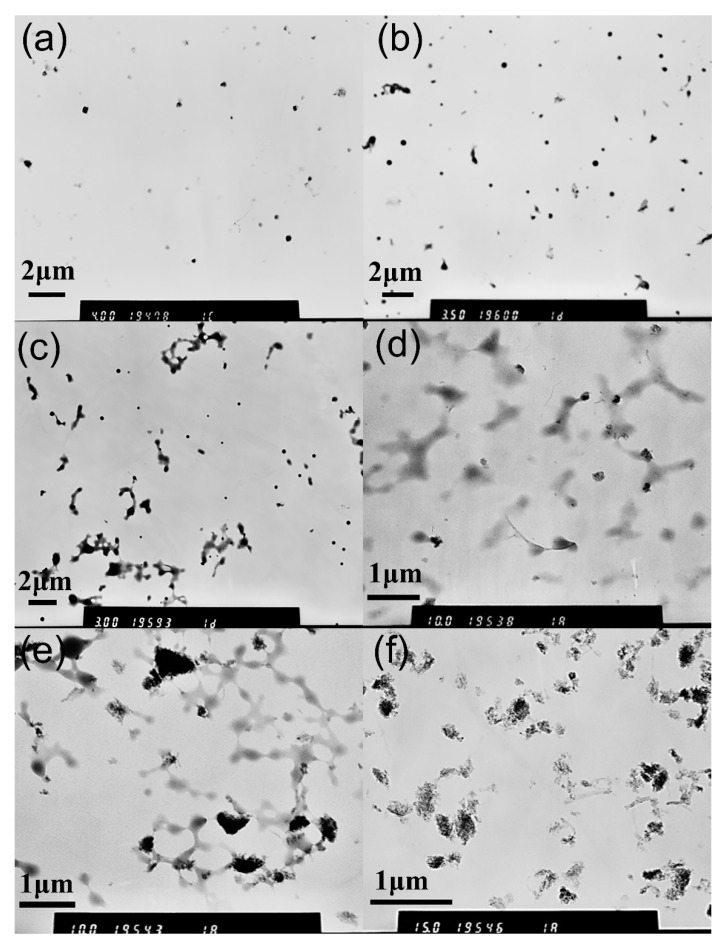
Transmission electron microscopy (TEM) images of drug-loaded methoxy polyethylene glycol-poly (D,L-lactic acid) (MPEG-PDLLA)/carbon nanotube (CNT) nanocomposites obtained at different mass ratios of carboxyl-modified multi-walled carbon nanotubes (c-MWCNTs)/MPEG-PDLLA: (**a**) 0; (**b**) 0.005:1; (**c**) 0.01:1; (**d**) 0.02:1; (**e**) 0.05:1; (**f**) 0.1:1.

To investigate the effects of different amounts of c-MWCNTs (diameter <8 nm, length 0.5~2 μm) on the structure of MPEG-PDLLA/c-MWCNT nanocomposites, we fixed the concentration of MPEG-PDLLA at 1.5 mg/mL, which is above the critical micelle concentration, and the mass ratios of c-MWCNTs relative to MPEG-PDLLA were 0, 0.005:1, 0.01:1, 0.02:1, 0.05:1 and 0.1:1, respectively. Transmission electron microscopy (TEM) was utilized to observe the morphology of MPEG-PDLLA/c-MWCNT nanocomposites. Figure 1 shows the morphology of the obtained MPEG-PDLLA/CNT nanocomposites and it can be seen from these images that the addition of c-MWCNTs has a great influence on the morphology of nanocomposites. As shown in Figure 1a, the MPEG-PDLLA molecules can easily self-assemble into spherical micelles with good dispersity and the average size is about 250 nm. When the mass ratio of c-MWCNTs/MPEG-PDLLA was 0.005:1, we found that some spherical micelles changed into rod-like micelles (Figure 1b). When the mass ratio of c-MWCNTs/MPEG-PDLLA further increased to 0.01:1, more spherical micelles changed into rod-like micelles, having a diameter of about 200 nm and a length of about 1 to 2 μm (Figure 1c). When the mass ratio of c-MWCNTs/MPEG-PDLLA reached up to 0.02:1, MPEG-PDLLA/c-MWCNT nanocomposites exhibited a rod-like morphology almost entirely, having a diameter of about 200 nm and a length of about 1 to 2 μm, as indicated in Figure 1d. However, in the case of the addition of c-MWCNTs at a ratio of 0.05:1, in addition to the rod-like micelles, some large irregular aggregates with a particle size of 200~500 nm also appeared in the TEM images (Figure 1e). When we continued to increase the mass ratio of c-MWCNTs/MPEG-PDLLA to 0.1:1, the TEM images showed such irregular aggregates almost completely (Figure 1f). Although the c-MWCNTs could not be observed directly in the TEM images, the length of the nanocomposites was within the length range of c-MWCNTs, and the diameter of the nanocomposite was close to the size of MPEG-PDLLA micelles with no c-MWCNT addition, which indicated that the c-MWCNTs were encapsulated in the formed MPEG-PDLLA/c-MWCNT supramolecular structures.

**Figure 2:**
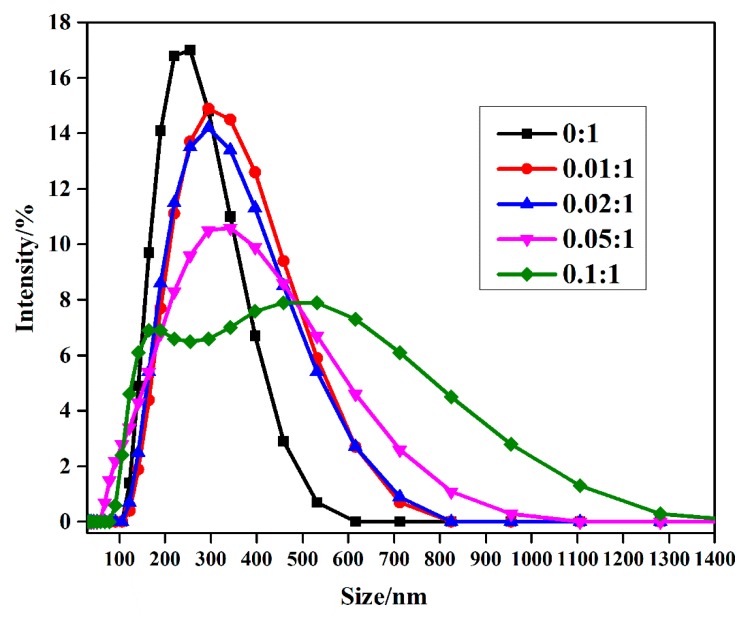
Size distributions of drug-loaded MPEG-PDLLA/c-MWCNT nanocomposites prepared from different amounts of c-MWCNTs added to the MPEG-PDLLA/c-MWCNTs mixed solutions (determined by dynamic light scattering (DLS) at 25 °C).

To obtain more intuitive size data of nanocomposites with different c-MWCNT additions, a dynamic light scattering (DLS) test was carried out to study the size and distribution of nanocomposites in water. The effect of c-MWCNT addition on the intensity-averaged diameter of MPEG-PDLLA/c-MWCNT nanocomposites is shown in Figure 2. DLS results showed that MPEG-PDLLA micelles had an average size of 250 nm at 25 °C. The effect of c-MWCNTs incorporated in the micelles on the size of hydrodynamic diameters had a change trend similar to that observed in the TEM images, where the average size of the nanocomposites increased with increasing c-MWCNT additions. It could also be seen from the DLS results that when the mass ratio of c-MWCNTs/MPEG-PDLLA increased from 0.05:1 to 0.1:1, the particle size distribution of the nanocomposites in the solution became larger, and the number of nanocomposites of the micron order also increased. Consequently, the TEM images and DLS data both indicate that MPEG-PDLLA/c-MWCNT nanocomposite structures were successfully prepared by the dialysis of MPEG-PDLLA and c-MWCNT mixed solutions against deionized water. Nanocomposites exhibiting the c-MWCNT addition-dependent structure transition demonstrate an effective method for the preparation of nanocomposite supramolecular structures.

**Figure 3:**
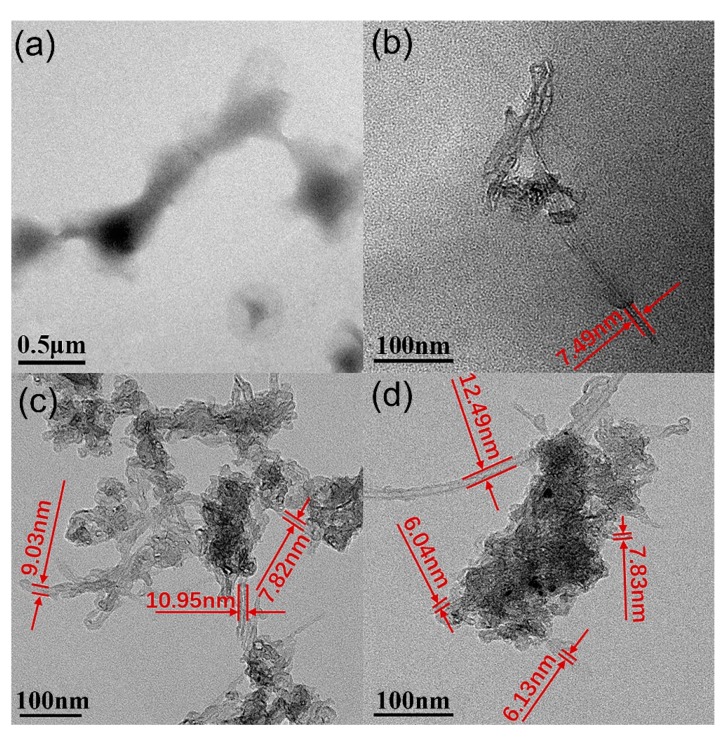
High-resolution transmission electron microscopy (HR-TEM) images of drug-loaded MPEG-PDLLA/c-MWCNT nanocomposites prepared at different mass ratios of c-MWCNTs/MPEG-PDLLA: (**a**,**b**) 0.02:1 and (**c**,**d**) 0.1:1.

High-resolution transmission electron microscopy (HR-TEM) analysis was also used to observe more details of the structure of MPEG-PDLLA/c-MWCNT nanocomposites and explore the interaction between the c-MWCNTs and MPEG-PDLLA. Figure 3 shows the HR-TEM images of MPEG-PDLLA/c-MWCNT nanocomposites with the mass ratios of c-MWCNTs/MPEG-PDLLA of 0.02:1 and 0.1:1. When a small amount of c-MWCNTs (MPEG-PDLLA/c-MWCNT ratio of 0.02:1) was added, c-MWCNTs were still not observed at a low magnification (Figure 3a). More closely observing the HR-TEM images at a high magnification, a c-MWCNT with a diameter of 7.49 nm was recognized in the residue of thermal degradation of nanocomposites, as indicated in Figure 3b, which confirmed that c-MWCNTs were encapsulated in the MPEG-PDLLA, forming rod-like micelles. In the case of a larger addition of c-MWCNTs (MPEG-PDLLA/c-MWCNT ratio of 0.1:1), some recognized c-MWCNTs were found to extend from the large aggregates and the diameter of individual c-MWCNTs was usually slightly larger than 8 nm, which was perhaps caused by the adsorption of MPEG-PDLLA on the surface of c-MWCNTs as indicated in Figure 3c. After careful observation of the HR-TEM images at high magnification, aggregates composed of c-MWCNTs and MPEG-PDLLA were well recognized, as shown in Figure 3d. Interestingly, c-MWCNTs, which had a relative larger diameter such as 12.49 nm, were not encapsulated in the aggregates.

**Figure 4:**
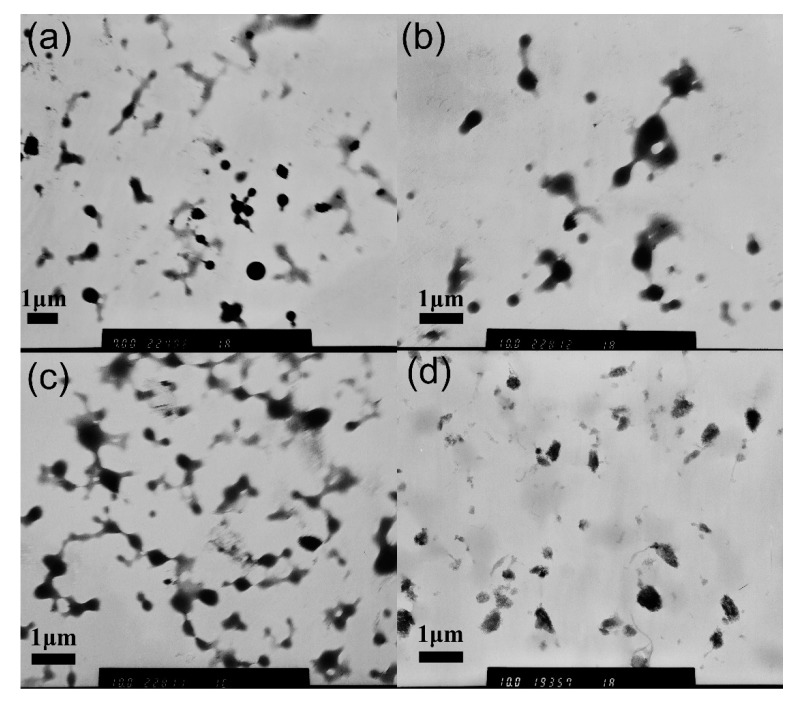
Transmission electron microscopy (TEM) images of mixing MPEG-PDLLA micelles with c-MWCNTs (addition at a mass ratio of c-MWCNTs/MPEG-PDLLA 0.1:1) for different times: (**a**) 0.5 h; (**b**) 2 h; (**c**) 6 h; (**d**) 24 h.

The results obtained from the TEM images indicated that a small addition of c-MWCNTs to MPEG-PDLLA could effectively induce the supramolecular structure transition from core–shell spherical micelles to rod-like micelles. However, it was uncertain whether the transition from rod-like micelles to large aggregates at high c-MWCNT additions was caused by the self-assembly of MPEG-PDLLA regulated by the dispersed c-MWCNTs, which may also be the structure formed by the MPEG-PDLLA adsorbing onto the undispersed carbon nanotube bundles. To explore the formation mechanism of these large aggregates, the dynamic mixing process of MPEG-PDLLA micelle solutions with c-MWCNTs was monitored by TEM. In this experience, MPEG-PDLLA with a fixed concentration of 1.5 mg/mL was the first to form micelles and then the c-MWCNT addition, added to form a mass ratio of 0.1:1 c-MWCNTs/MPEG-PDLLA, was mixed with the MPEG-PDLLA micelles. Figure 4 shows the TEM images of MPEG-PDLLA/c-MWCNT nanocomposites prepared with different mixing times between the MPEG-PDLLA micelles and c-MWCNTs. When the mixing time was 30 min, as shown in Figure 4a, some spherical micelles changed into rod-like micelles, which was similar to the morphology observed with the low c-MWCNT addition, as indicated in Figure 1c. Furthermore, no carbon nanotube bundles were found. When the mixing time was extended from 30 min to 24 h, the morphology of the formed MPEG-PDLLA/c-MWCNT nanocomposites gradually changed from a rod-like morphology to irregular aggregates, and this morphology changing trend was similar to that of the nanocomposites prepared by the dialysis of c-MWCNTs/MPEG-PDLLA solutions against aqueous water with the c-MWCNT addition ranging from low to high. The time-dependent morphology change results confirmed that the irregular aggregates were formed by the interaction between MPEG-PDLLA and c-MWCNTs, rather than the self-agglomeration of c-MWCNTs.

**Figure 5:**
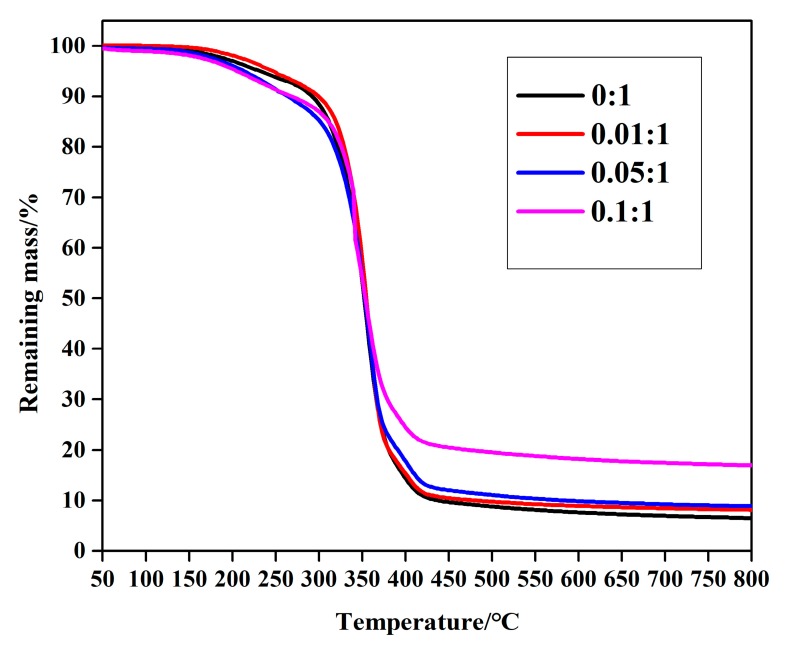
Thermal gravimetric analysis (TGA) curves (50–800 °C) of drug-loaded MPEG-PDLLA/c-MWCNT nanocomposites prepared from different amounts of c-MWCNTs added to the MPEG-PDLLA/c-MWCNT mixed solutions.

Thermal gravimetric analysis (TGA) was further used to directly examine the content of c-MWCNTs that was involved in the formation of MPEG-PDLLA/c-MWCNT nanocomposites. The changes in the weight of MPEG-PDLLA micelles and MPEG-PDLLA/c-MWCNT nanocomposites under a nitrogen atmosphere as a function of temperature are given in Figure 5. As can be seen from Figure 5, the final remaining mass of samples after degradation was complete increased with an increase in the amount of c-MWCNT additions. The final residual mass increased with the increasing MWCNT additions because c-MWCNTs have high thermal stability and do not decompose in the test temperature range.

**Figure 6:**
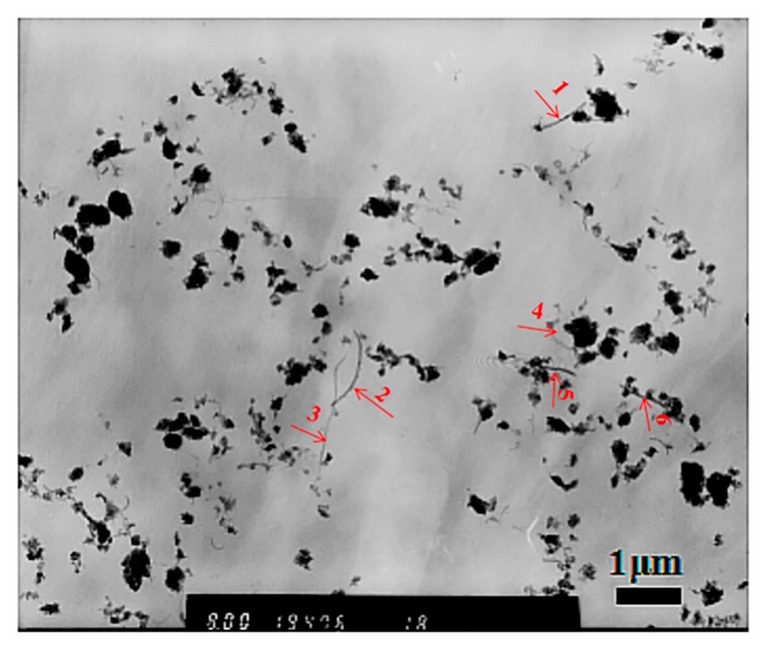
Transmission electron microscopy (TEM) images of drug-loaded MPEG-PDLLA/c-MWCNT nanocomposites prepared with a mass ratio of c-MWCNTs/MPEG-PDLLA of 0.1:1 (tube diameter <8 nm, length 0.5~2 μm).

The above results demonstrated that the c-MWCNTs with a diameter <8 nm participated in the self-assembly process of MPEG-PDLLA and played a regulator role in the assembly into supramolecular structures. Moreover, a careful examination of the TEM images revealed that some c-MWCNTs with large diameters were not encapsulated in the aggregates formed at a high addition of c-MWCNTs, or that they could not be recognized. To further confirm this phenomenon, we carefully examined the TEM images in a large range in the case of a high addition of c-MWCNTs (the mass ratio of c-MWCNTs/MPEG-PDLLA was 0.1:1). As indicated in Figure 6, some well-dispersed single c-MWCNTs could be clearly recognized and they did not seem to participate in the self-assembly of MPEG-PDLLA. By measuring their diameter (Table 1), we found that the diameter of these c-MWCNTs ranged from 14 to 30 nm, which is far larger than 8 nm. These obtained results suggest that the diameter size of c-MWCNTs may play an important role in the cooperative self-assembly of c-MWCNTs and MPEG-PDLLA.

**Figure 7:**
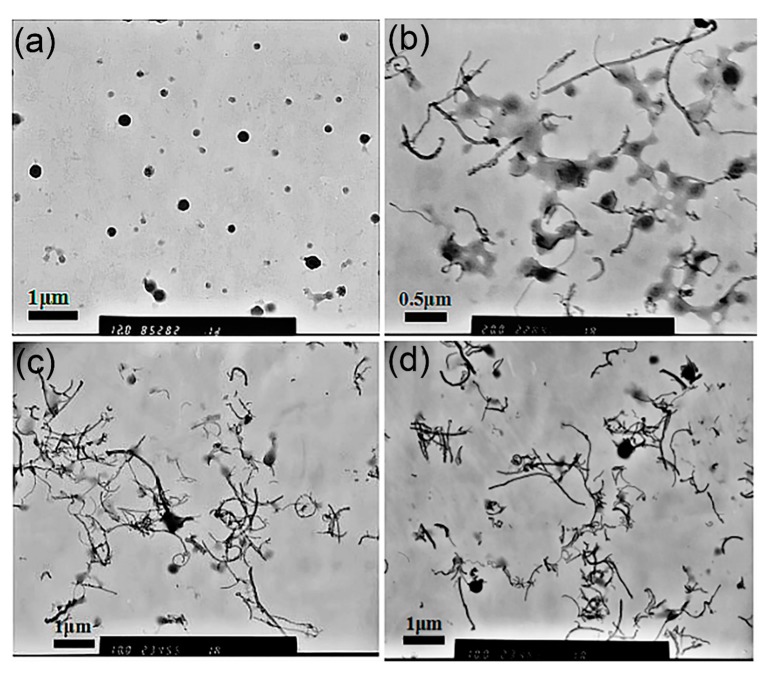
Transmission electron microscopy (TEM) images of drug-loaded MPEG-PDLLA/c-MWCNT nanocomposites obtained at different mass ratios of c-MWCNTs (tube diameter >30 nm, length 0.5~2 μm) to MPEG-PDLLA: (**a**) 0; (**b**) 0.05:1; (**c**) 0.2:1; (**d**) 0.3:1.

**Figure 8:**
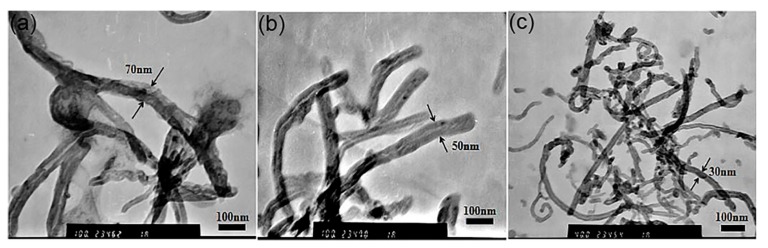
Transmission electron microscopy (TEM) images of drug-loaded MPEG-PDLLA/c-MWCNT nanocomposites obtained at different mass ratios of c-MWCNTs (tube diameter >30 nm, length 0.5~2 μm) to MPEG-PDLLA: (**a**) 0.2:1; (**b**) 0.3:1 and (**c**) 1:0.

To explore whether this different assembly behavior of MPEG-PDLLA was caused by the diameter of c-MWCNTs, c-MWCNTs (tube diameter 30~50 nm, length 0.5~2 μm) were also chosen for the preparation of MPEG-PDLLA/c-MWCNT nanocomposites. As shown in Figure 7, the morphology of MPEG-PDLLA/c-MWCNT nanocomposites fabricated with c-MWCNTs (tube diameter 30~50 nm, length 0.5~2 μm) was quite different from that of the previous MPEG-PDLLA/c-MWCNT nanocomposites (tube diameter <8 nm, length 0.5~2 μm), as indicated in Figure 1. When adding c-MWCNTs (tube diameter 30~50 nm, length 0.5~2 μm) to the MPEG-PDLLA solution, the MPEG-PDLLA micelle structure gradually disappeared and the c-MWCNTs exhibited a well-dispersed individual nanotube state. In the case of a small addition of c-MWCNTs (Figure 8b), some large micelles coexisted with the dispersive c-MWCNTs, as shown in the TEM images. With the increase of the c-MWCNT addition, these micelles disappeared gradually, and only well-dispersed c-MWCNTs could be found in the TEM images at high c-MWCNT additions (Figure 7c,d).

Upon observing the TEM images more closely, we found that the diameter of c-MWCNTs increased with the decreasing addition in the MPEG-PDLLA solutions, as shown in Figure 8. When the mass ratio of c-MWCNTs/MPEG-PDLLA increased to 0.2:1 and 0.3:1, the diameters of MPEG-PDLLA/c-MWCNT nanocomposites were about 70 nm and 50 nm, respectively (Figure 8a,b), as compared to the 30 nm of the pure c-MWCNTs (Figure 8c). These results indicated that the MPEG-PDLLA was mainly adsorbed on the surface of c-MWCNTs (tube diameter 30~50 nm, length 0.5~2 μm), forming an adsorption layer, which resulted in the increasing diameter and the good dispersion of c-MWCNTs.

### 3.2. Proposed Possible Structures for MPEG-PDLLA/c-MWCNT Nanocomposites

By comparing the TEM images of MPEG-PDLLA/c-MWCNT nanocomposites, we found that the addition amount and diameter of c-MWCNTs have different effects on the formation of nanocomposites. The morphology of nanocomposites can transform from core–shell spherical micelles to rod-like micelles or large composite aggregate structures to encapsulate c-MWCNTs upon the increasing addition of c-MWCNTs with a small diameter. Meanwhile, when the c-MWCNTs have a large diameter, the amphiphilic copolymer is mainly adsorbed on the surface of c-MWCNTs, causing the c-MWCNTs to exhibit good dispersion.

When the mixtures of c-MWCNTs and MPEG-PDLLA were dialyzed against aqueous water, the hydrophobic segment of MPEG-PDLLA generally anchored the chain to the surface of c-MWCNTs via hydrophobic interactions. c-MWCNTs with a small diameter (tube diameter <8 nm, length 0.5~2 μm) can only provide a narrow surface on which the PDLLA segments can adsorb. In this case, it is very difficult for PDLLA segments and c-MWCNTs to fully contact and interact. Thus, most of the hydrophobic chain dangles outside the carbon nanotubes along with the hydrophilic chain and constituted a new “temporary shape amphiphile”. Then this new build block still can interact with the MPEG-PDLLA molecules and they can cooperatively assemble into energetically stable nanocomposite supramolecular structures in an aqueous solution. When the addition amount of c-MWCNTs (tube diameter <8 nm, length 0.5~2 μm) was small, the sufficient MPEG-PDLLA and MPEG-PDLLA-CNT amphiphiles would cooperatively assemble into a rod-like aggregate structure in which c-MWCNTs play a role as a template (Figure 1b–d). When the addition amount of c-MWCNTs was high, if MPEG-PDLLA still formed rod-like micelles directed by single c-MWCNTs, due to the lack of sufficient MPEG-PDLLA molecules, some hydrophobic segments of MPEG-PDLLA and c-MWCNTs would both be exposed in the solution, making the structures unstable. In this case, some small aggregates might be formed between c-MWCNTs and then MPEG-PDLLA would form new larger composite aggregates around the c-MWCNT aggregates to completely encapsulate the c-MWCNTs (Figure 1e,f).

When c-MWCNTs (tube diameter 30~50 nm, length 0.5~2 μm) were used to mix with MPEG-PDLLA, the large diameter of c-MWCNTs provided a wide graphene band-like substrate for PDLLA anchoring. In this case, the PDLLA segment could not only have a strong interaction with the surface of c-MWCNTs, but a strong intermolecular interaction could also occur between the PDLLA segments adsorbed and arranged on the surface of c-MWCNTs. In addition, the hydrophilic MPEG segments were dangled outside the c-MWCNTs, which also enhanced the PDLLA–c-MWCNT interaction and the interaction of the PDLLA segments adsorbed on the surface of CNTs. These combined effects lead to the good dispersion of c-MWCNTs even under the conditions of a high addition of c-MWCNTs, as indicated in Figure 7.

**Figure 9:**
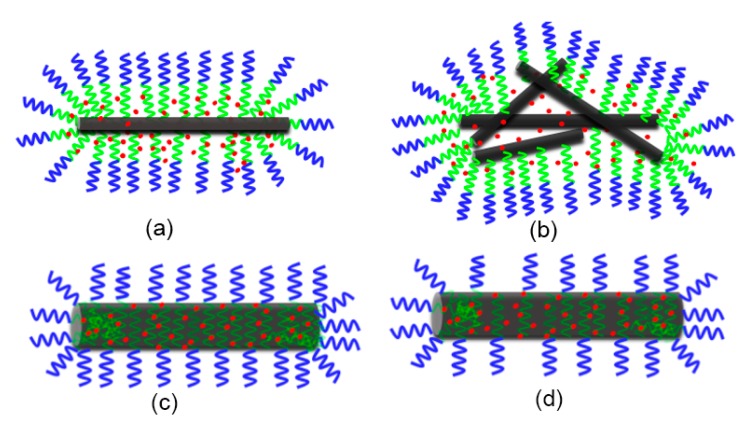
Schematic representation of the possible formation structures of drug-loaded MPEG-PDLLA/c-MWCNT nanocomposites prepared with different amounts of c-MWCNTs added to the MPEG-PDLLA solutions: (**a**) a low addition of c-MWCNTs with a small diameter; (**b**) a high addition of c-MWCNTs with a small diameter; (**c**) a low addition of c-MWCNTs with a large diameter; (**d**) a high addition of c-MWCNTs with a large diameter.

According to the above analysis, a schematic diagram of how c-MWCNTs combine with MPEG-PDLLA to form MPEG-PDLLA/c-MWCNT nanocomposites could be outlined, as indicated in Figure 9.

### 3.3. Stability of MPEG-PDLLA/c-MWCNT Nanocomposites

**Figure 10:**
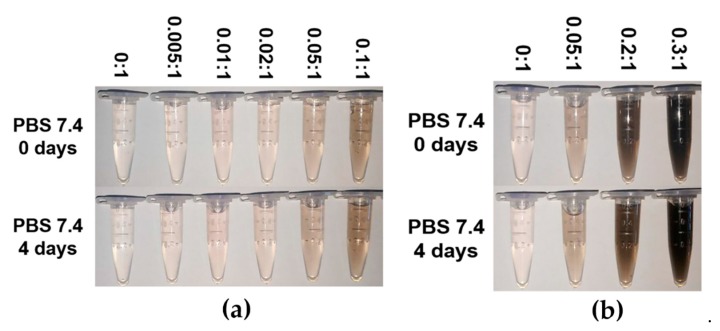
Dispersion stability of drug-loaded MPEG-PDLLA/c-MWCNT nanocomposites prepared from different amounts of c-MWCNTs added to the MPEG-PDLLA solutions in phosphate buffer solution (PBS): (**a**) c-MWCNTs with a small diameter (tube diameter <8 nm, length 0.5~2 μm) and (**b**) c-MWCNTs with a large diameter (tube diameter 30~50 nm, length 0.5~2 μm).

The dispersion stability of the obtained MPEG-PDLLA/c-MWCNT nanocomposites was evaluated using a digital camera. Optical images of the dispersion of MPEG-PDLLA/c-MWCNT nanocomposites with different c-MWCNT additions in phosphate butter solution are shown in Figure 10. All the MPEG-PDLLA/c-MWCNT nanocomposites showed stable dispersion in PBS and showed no occurrence of any observed precipitation in four days. As a hydrophilic non-ionic polymer, PEG segments could form a hydrophilic shell on the MPEG-PDLLA/c-MWCNT nanocomposites, which improved the dispersion stability and provided the bioavailability of these nanocomposites for biomedical applications. These results obtained from the TEM images and the dispersion stability analysis indicated that the MPEG-PDLLA/c-MWCNT nanocomposites exhibited a well-defined stable nanostructure, representing a crucial structure feature for these functional nanostructures to be used in biomedical field.

### 3.4. Drug Loading and In Vitro Release Studies

To explore the potential use of MPEG-PDLLA/c-MWCNT nanocomposites as carriers for chemotherapeutic drugs, the drug-loading ability and release behavior were studied. Table 2 and Table 3 show the drug loading of the MPEG-PDLLA/c-MWCNT nanocomposites with different diameters of c-MWCNTs. It was found that the addition of c-MWCNTs increased the drug loading of nanocomposites. The effect of c-MWCNT addition on the drug loading of amphiphilic block polymer MPEG-PDLLA was mainly due to its modification of the hydrophobic segment of MPEG-PDLLA. In the MPEG-PDLLA/c-MWCNT nanocomposites, that the MPEG-PDLLA adsorbed on the surface of c-MWCNTs could expand the space between the hydrophobic segments of MPEG-PDLLA compared with that in the pure MPEG-PDLLA micelles, and created a larger hydrophobic space. Thus, the nanocomposites exhibited increased interact sites with hydrophobic drugs and could encapsulate more drugs.

**Figure 11:**
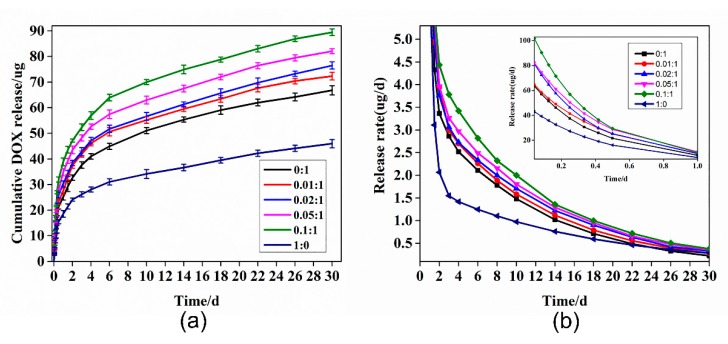
Release profiles of doxorubicin (DOX) from DOX-loaded nanocomposites prepared from the different addition amounts of c-MWCNTs (tube diameter <8 nm, length 0.5~2 μm) in the MPEG-PDLLA/c-MWCNT mixed solutions: (**a**) cumulative DOX release from drug-loaded micelles and (**b**) the DOX release rate. Data are shown as means ± S.D. (*n* = 6).

**Figure 12:**
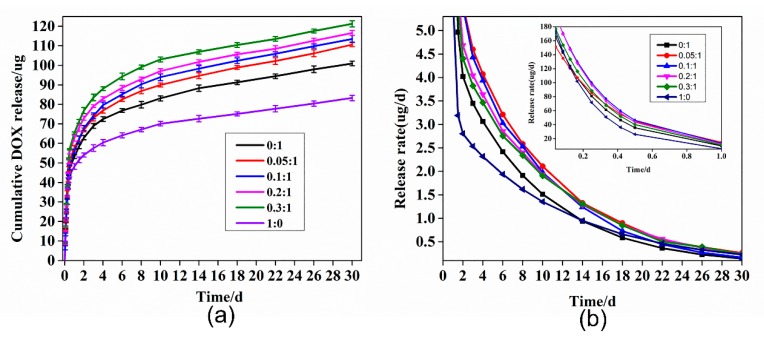
Release profiles of DOX from DOX-loaded nanocomposites prepared from the different addition amounts of c-MWCNTs (tube diameter 30~50 nm, length 0.5~2 μm) in the MPEG-PDLLA/c-MWCNT mixed solutions: (**a**) cumulative DOX release from drug-loaded micelles and (**b**) the DOX release rate. Data are shown as means ± S.D. (*n* = 6).

The in vitro drug release profiles of MPEG-PDLLA/c-MWCNT nanocomposites with different addition amounts of c-MWCNTs were studied in PBS at 37 °C and pH 7.4, mimicking the temperature and pH of the human body. As indicated in Figure 11 and Figure 12, MPEG-PDLLA/c-MWCNT nanocomposites at all tested groups showed an initial burst release of loaded DOX in the first two days, releasing almost 60% of the total loaded DOX, then continuing releasing at a slightly slower steady rate for 30 days.

The rate of drug release from the nanocomposites was calculated according to the drug release curve (Figure 11 and Figure 12). It can be seen from the data that when the addition amounts of c-MWCNTs (tube diameter <8 nm, length 0.5~2 μm) increased, the sustained release rate of the drug also increased. The initial burst release phenomenon may be due to the release of DOX that adsorbed in the shell. During the steady release phase, DOX that was loaded in the inner hydrophobic core of micelles was released, causing a slow release rate. During the drug release process, the morphology of nanocomposites has an important effect on the drug release rate. Although c-MWCNT additions (tube diameter <8 nm, length 0.5~2 μm) increased the spacing between hydrophobic segments of MPEG-PDLLA for drug loading, it also weakened the interaction force between hydrophobic segments of MPEG-PDLLA, leading to an accelerated release rate of the drug. Therefore, the greater the addition amount of c-MWCNTs, the faster the drug release rate that can be achieved.

However, the change trend of the release rate from the MPEG-PDLLA/c-MWCNT nanocomposites with a diameter of 30~50 nm was different from that of MPEG-PDLLA/c-MWCNT nanocomposites with a diameter less than 8 nm, as indicated in Figure 12. With the addition of c-MWCNTs, all the sustained drug release rates also increased compared with that of the pure MPEG-PDLLA micelles. However, when the mass ratio of c-MWCNTs to MPEG-PDLLA was 0.05:1, the sustained release rate of the composite drug carrier was the fastest. When the addition amounts of c-MWCNTs continued to increase, the sustained drug release rate began to decrease. This phenomenon can be ascribed to the fact that the hydrophobic segments of MPEG-PDLLA fully interacted with c-MWCNTs (tube diameter 30~50 nm, length 0.5~2 μm), forming an adsorption layer on the surface of c-MWCNTs. The distance between the hydrophobic segments was larger compared to that of the micelles with no c-MWCNT addition, which weakened the interaction force with the drug and thus accelerated the drug release rate. When increasing the addition amount of c-MWCNTs, the distance between the hydrophobic segments of MPEG-PDLLA adsorbed on the surface of c-MWCNTs also increased. In this case, a portion of the drug was loaded onto the surface of c-MWCNTs and the stronger interaction force between the DOX and c-MWCNTs caused the drug release rate to decrease.

## 4. Conclusions

In conclusion, we explored the effect of adding c-MWCNTs to methoxy polyethylene glycol-poly (D, L-lactic acid) (MPEG-PDLLA) micelles on the structure and corresponding drug release. The cooperative self-assembly of MPEG-PDLLA and c-MWCNTs with a diameter <8 nm can change from core–shell spherical micelles to rod-like micelles and then to large composite aggregates by increasing the addition amount of c-MWCNTs. The morphological transition mechanism of the cooperatively self-assembled structures was proposed based on the TEM results. Due to the narrower size of diameter, c-MWCNTs with a diameter <8 nm could not provide space enough to allow the MPEG-PDLLA to fully anchor on the surface of CNTs, so most of the hydrophobic chain dangled outside the carbon nanotubes along with the hydrophilic chain and constituted a new “temporary shape amphiphile”. These “temporary shape amphiphiles” and MPEG-PDLLA could cooperatively assemble into different supramolecular aggregates, providing an adequate hydrophobic cargo to adopt the c-MWCNTs according to the addition amount of c-MWCNTs. However, in the case of adding c-MWCNTs with a diameter of 30~50 nm to the MPEG-PDLLA solutions, the wide diameter of c-MWCNTs allowed the PDLLA segments of MPEG-PDLLA to fully anchor onto the surface of CNTs and screen the hydrophobic interactions among the CNTs, while the hydrophilic block dangled out of the CNTs and enabled their good dispersion. Furthermore, as-prepared MPEG-PDLLA/c-MWCNT nanocomposites could be used as drug carriers to control the drug loading and release. The results obtained provide new insights into the understanding of the role of carbon nanotubes in adjusting the self-assembly behavior and drug release of amphiphilic block copolymers and further demonstrate the future potential for the controllable fabrication of functional nanostructures for biomedical applications.

## Figures and Tables

**Table 1:** Diameter of c-MWCNTs in drug-loaded MPEG-PDLLA/c-MWCNT nanocomposites prepared with a mass ratio of c-MWCNTs/MPEG-PDLLA of 0.1:1 (tube diameter <8 nm, length 0.5~2 μm).

Number	Size/nm
1	30
2	22
3	15
4	14
5	23
6	20

**Table 2:** Drug entrapment (DE%) of drug-loaded MPEG-PDLLA/c-MWCNT nanocomposites prepared from the MPEG-PDLLA/c-MWCNTs mixed solutions with different c-MWCNT (tube diameter <8 nm, length 0.5~2 μm) additions.

c-MWCNT Addition	Drug Entrapment (DE%)
0:1	4.35 ± 0.20
0.01:1	4.72 ± 0.25
0.02:1	4.51 ± 0.15
0.05:1	5.38 ± 0.32
0.1:1	6.22 ± 0.35
1:0	2.53 ± 0.10

**Table 3:** Drug entrapment (DE%) of drug-loaded MPEG-PDLLA/c-MWCNT nanocomposites prepared from the MPEG-PDLLA/c-MWCNTs mixed solutions with different c-MWCNT (tube diameter >30 nm, length 0.5~2 μm) additions.

c-MWCNT Addition	Drug Entrapment (DE%)
0:1	4.35 ± 0.20
0.05:1	4.97 ± 0.27
0.1:1	5.71 ± 0.31
0.2:1	5.13 ± 0.23
0.3:1	6.86 ± 0.36
1:0	3.65 ± 0.13
